# Case Report: A Novel Reconstruction Method (Itihaas’s Anastomosis) for Proximal Gastrectomy

**DOI:** 10.3390/healthcare13141663

**Published:** 2025-07-10

**Authors:** Birendra Kumar Sah, Zhenjia Yu, Chen Li, Zhenggang Zhu

**Affiliations:** Gastrointestinal Surgery Unit, Department of General Surgery, Ruijin Hospital, Shanghai Jiao Tong University School of Medicine, 197 Ruijin Er Road, Shanghai 200025, China; 15000029773@163.com (Z.Y.); lc10897@rjh.com.cn (C.L.); zzg@rjh.com.cn (Z.Z.)

**Keywords:** case report, proximal gastrectomy, Itihaas’s Anastomosis

## Abstract

This study introduces a novel reconstruction method (Itihaas’s Anastomosis) for the upper gastrointestinal (UGI) tract following proximal gastrectomy, designed to mitigate the severity of acid reflux syndrome, a frequent postoperative complication. The procedure comprises three side-to-side anastomoses: esophago-gastrostomy, gastro-jejunostomy, and jejuno-jejunostomy. The esophago-gastrostomy anastomosis aims to prevent direct reflux of gastric contents into the esophagus by creating a fundus-like structure, which also facilitates future endoscopic procedures. The gastro-jejunostomy reduces acid reflux by diverting gastric acid to the jejunum for further neutralization, while the jejuno-jejunostomy prevents bile and pancreatic juice reflux into the stomach. A 75-year-old male with adenocarcinoma of the upper stomach underwent this surgical procedure. Postoperative outcomes showed no major complications, with smooth oral contrast passage and no evidence of anastomotic leaks. The patient was discharged after resuming consumption of semi-solid food and experienced no signs of reflux. Itihaas’s Anastomosis represents a novel technical approach that theoretically may reduce acid reflux following proximal gastrectomy. Initial case experience suggests technical feasibility, though all claimed benefits remain theoretical without objective validation. Long-term outcomes, anti-reflux efficacy, and comparative effectiveness require validation through systematic case series with objective assessments.

## 1. Introduction

**Figure 1:**
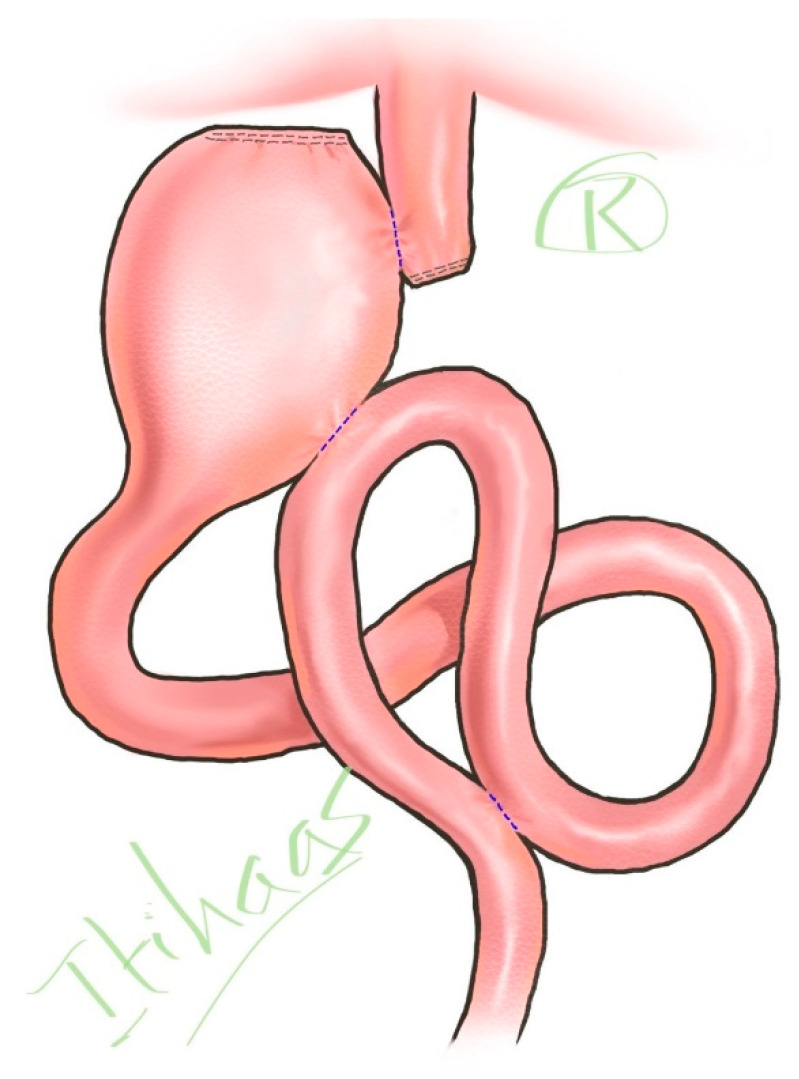
Itihaas’s Anastomosis for proximal gastrectomy.

We introduce a novel reconstruction method (Itihaas’s Anastomosis) for the upper gastrointestinal (GI) tract following proximal gastrectomy, aimed at decreasing the severity of acid reflux syndrome, a common post-surgical complication. The procedure involves performing three anastomoses (Figure 1).

This diagram focuses primarily on the anastomotic configurations rather than detailed biliary anatomy, as surgeons in this specialty are generally familiar with standard biliary and pancreatic anatomy. Future iterations may include enhanced anatomical detail for broader readership.

1. Esophago-Gastrostomy: The first side-to-side anastomosis of esophago-gastrostomy is hypothesized to be beneficial in theoretically preventing the direct reflux of gastric juice or food into the esophagus. This is due to the side-to-side anastomosis creating a fundus-like structure in the stomach, confirmed by radiological imaging after oral contrast ingestion. This method also preserves the natural upper gastrointestinal tract, which is beneficial for patients who may need endoscopic retrograde cholangiopancreatography (ERCP) for future biliary diseases.
2. Gastro-Jejunostomy: The second anastomosis is a side-to-side gastro-jejunostomy aimed at theoretically reducing acid reflux. The hypothesis is that bypassing acid to the jejunum allows it to be further neutralized, decreasing the acid concentration in the stomach and preventing reflux into the esophagus.
3. Jejuno-Jejunostomy: To theoretically prevent bile and pancreatic juice reflux into the remnant stomach, a side-to-side jejuno-jejunostomy is performed.

This combination of anastomoses represents a potential alternative that requires validation for reducing acid reflux syndrome and improving postoperative quality of life for patients undergoing proximal gastrectomy.

Limitations and Study Rationale: This case report presents a preliminary technical description of a novel reconstruction method. All stated benefits are theoretical and require validation through well-designed prospective studies. This report serves as the first step before formal comparative studies.

### 1.1. Patient Information

A 75-year-old male presented with upper abdominal distension and mild pain. He had no history of chronic illness, including hypertension, diabetes, cardiac diseases, asthma, or cerebrovascular diseases. There was no family history of malignant diseases, and he had no past mental illness or genetic disorders. The patient had no previous surgeries or interventional therapies, including endoscopic treatments. He was a mild alcoholic and cigarette smoker, consuming about 10 cigarettes per day.

### 1.2. Clinical Findings

The patient was skinny but had a normal nutrition level, with no signs of malnutrition. He had normal cognitive function and was able to respond independently to medical queries. Vital signs, including blood pressure, oxygen saturation, heart rate, and respiratory rate, were normal. No enlarged lymph nodes were palpable in superficial regions. Abdominal examination revealed a flat abdomen with no palpable mass or tenderness, and normal bowel sounds were present. Digital rectal examination found no mass in the rectum or pelvis, and there was no blood on the glove.

### 1.3. Diagnostic Assessment

Gastroscopy and tissue biopsy diagnosed the patient with adenocarcinoma of the upper gastric region, below the cardia on the posterior wall near the lesser curvature. The lesion was a superficial ulcerative, approximately 25 mm in diameter, classified as Borrmann II type cancer. Abdominal CT with contrast confirmed the lesion in the upper stomach, with no evidence of extraserosal infiltration or grossly enlarged lymph nodes. There were no distant metastases or retroperitoneal lymph nodes detected, and the cancer was staged as clinical stage II. Blood tests, including tumor markers (CA-125, CA-199, CA-724, AFP, and CEA), were normal. Routine blood tests showed no anemia, and hepatic and renal function tests were normal. EKG, cardiac sonography, and pulmonary function tests indicated acceptable heart and lung function for major surgery.

### 1.4. Intervention

A radical proximal gastrectomy with adequate lymph node dissection was proposed during the multidisciplinary team discussion. A nasoenteric nutritional tube was inserted into the stomach before surgery on the same day, with the intention to place it in the jejunum at least 20 cm distal to the last anastomosis during the surgery. The patient was operated on under general anesthesia in a supine position, and a conventional open surgery was performed through laparotomy.

After initial exploration of the abdomen, no peritoneal or distant organ metastases were found in the abdominal or pelvic cavities. There was no ascites. The tumor was confined to the upper gastric region with no sign of extraserosal invasion. No obvious mass was present, but mild thickening of the gastric wall was palpated in the lesser curvature below the cardia. A few lymph nodes were palpated on the right side of the tumor in the No. 1 and No. 3 lymph node areas according to the distribution of stomach cancer.

The surgery was performed using a standard technique for curative gastrectomy with D2 lymph node dissection for proximal gastric cancer. Key steps included the following:

1. The gastro-colonic ligament was divided starting from the right colon liver flexure toward the left. The greater omentum was resected while preserving the blood vessel arch in the greater curvature of the stomach until it was just proximal to the gastric antrum. Blood vessels were divided, and complete resection of the omentum was performed proximally. Lymph node dissection was carried out around the left epiploic vessels. The gastro-splenic ligaments were divided, and all gastric short arteries were ligated. All surrounding fatty tissues and lymph nodes were dissected up to the lower abdominal segment of the esophagus.
2. The esophagus was freed by dissecting the surrounding tissues and diaphragmatic crests on the left side. Lymph node dissection was performed in the hepatic-duodenal ligament, dissecting No. 12 and No. 8 lymph nodes, with careful preservation of the right gastric vessels. The lesser omentum and gastric hepatic ligament were dissected proximally, continuing the dissection of lymph nodes in the lesser curvature of the stomach up to the right side of the cardia and abdominal segment of the esophagus.
3. After careful evaluation of safe margins, the stomach was divided at the body with an automatic linear stapler and dissector. After holding the stomach upward, standard lymph node dissection was performed along the superior border of the pancreas, dissecting all lymph nodes in the area including, but not limited to, No. 11p, 11d, No. 9, and No. 7 LNs. The left gastric vessels were ligated and divided at their origin at the celiac trunk. Posterior gastric vessels were ligated and divided, and the stomach was freed posteriorly. Adequate length of the esophagus was freed from adjacent ligaments via a transhiatal approach, approximately 7–8 cm of the lower esophagus.
4. After careful examination of the tumor, a proximal margin was marked in the esophagus. The first assistant pulled the proximal stomach to the left side without touching the tumor, providing clear exposure of the lower esophagus. The surgeon made a small hole in the anterior wall of the stomach adjacent to the greater curvature and another aperture on the right side of the esophagus, slightly anteriorly above the marked safe margin. Prior to reconstruction, tumor margins were assessed using preoperative endoscopic carbon nanoparticle injection for precise margin identification. A side-to-side gastro-esophageal anastomosis was performed with an automatic linear stapler and dissector. After careful examination of the reconstructed cavity through the common hole, the safe margin of the tumor was reconfirmed, and no signs of bleeding were present. The esophagus was then divided with an automatic linear stapler and dissector, and the en bloc specimen was sent for pathological examination. The proximal border (esophagus) of the specimen was sent for a frozen section. Later, it was confirmed that there was no tumor infiltration. The common aperture was closed with continuous sutures using 3–0 Vicryl (polyglactin 910) sutures (Ethicon, Johnson & Johnson, New Brunswick, NJ, USA)threads.
5. A gastro-jejunal anastomosis was performed at the bottom of the stomach, on the posterior wall near the greater curvature. Approximately 15 cm from the ligament of Treitz, a jejuno-jejunal anastomosis was performed with the distal jejunum, approximately 20 cm distal from the gastro-jejunostomy. Both anastomoses were performed with an automatic linear stapler and dissector, and the common aperture was closed with continuous sutures using 3–0 Vicryl threads after careful examination for any intramural bleeding.

Technical Considerations: Special attention was paid to securing all anastomoses to prevent potential herniation. The gastro-jejunostomy was positioned and secured to minimize risk of displacement, and adequate hiatal repair was performed to prevent small bowel herniation through the hiatus.

Two abdominal drainage tubes were placed: one posteriorly to the esophago-gastrostomy anastomosis and another close to the gastro-jejunostomy. The tip of the nasoenteric tube was then inserted up to 20 cm distal to the jejuno-jejunostomy by palpation and with assistance from the surgeon, after which it was securely fixed at the nose.

### 1.5. Follow-Up and Outcomes

The patient was monitored in the ward for 15 days. No major postoperative complications were observed, including abdominal bleeding, infection, or anastomotic leak. The patient passed gas on the third day and had their first bowel movement on the fourth day. There was no nausea, vomiting, abdominal distension, or abdominal pain reported. The patient began drinking liquids on the third day, started consuming liquid food on the ninth day, and moved to semi-solid food by the eleventh day. They received normal saline (250 mL) on the third postoperative day and enteral nutrition (500 mL plus 25 mL of normal saline) via a nasoenteric tube on the fifth day, increasing to 1000 mL plus 250 mL of normal saline on the ninth day. The patient tolerated the food well during the immediate postoperative period, with no complaints of nausea, vomiting, or abdominal pain, and passed gas and stool in a timely manner. However, long-term reflux assessment and quality of life evaluation remain to be determined. No objective reflux assessments were performed in this case. Future cases will include endoscopic evaluation, pressure measurements (manometry), and validated quality of life questionnaires to provide concrete evidence of anti-reflux efficacy.

The patient had a mild fever, with a maximum temperature of 38.2 °C or below, for five days post-surgery. A CT scan diagnosed atelectasis in both lower lobes of the lungs, with no abnormal findings in the abdominal cavity or surgical site. Bacterial and fungal cultures revealed no pulmonary or abdominal infection. The surgical wound healed without any infection.

A postoperative oral contrast study showed smooth passage of contrast from the esophagus mainly to the stomach and partially to the jejunum, with no reflux from the stomach to the esophagus or from the jejunum to the stomach. Contrast passed smoothly from the jejunum into the distal jejunum. A plain CT scan taken 4 h after the oral contrast showed no signs of anastomotic leak, and the contrast was present in the colon, mainly in the transverse colon and distally to the rectum. The patient passed stools slightly more than usual in the evening due to the hyperosmotic nature of the oral contrast. Drainage tubes were removed on the 15th day for intentional observation, as this was the first case of this type of anastomotic procedure. The patient was discharged on the 16th day and asked to return for follow-up one month after the operation date.

The intentionally prolonged hospital stay (15 days) was implemented as a conservative approach for this first case using the novel technique, allowing for careful monitoring of oral intake and early complication detection. It is worth mentioning that the intentional delay in oral food intake was mainly to ensure a smooth recovery for this first anastomotic case and as a preventive or conservative approach due to the mild fever and mild elevation of white blood cells and neutrophils. Generally, patients at our center start oral water intake on the second postoperative day, liquid food on the fourth day, and semi-solid food on the sixth day. If they tolerate the food well with no signs of fever and normal white blood cell counts, they are discharged on the eighth day.

Postoperative pathological reports confirmed intestinal type gastric adenocarcinoma with subserosal invasion and no lymph node metastases (0/24 lymph nodes examined). The pathological stage was pT3N0M0, Stage IIA, with negative resection margins both proximally and distally. The D2 lymphadenectomy yielded an adequate lymph node harvest of 24 nodes, meeting standard oncological requirements.

## 2. Discussion

Study Limitations: This case report presents several important limitations. First, reflux symptoms cannot be adequately assessed in the immediate postoperative period, making any claims about reduced reflux theoretical. We currently have no concrete objective evidence that our ‘neo-fundus’ recreates the natural anti-reflux barrier or angle of His—this case description presents a novel method based on our center’s experience, and all assumptions are yet to be validated. Second, long-term outcomes including weight loss, dietary intake, and quality of life cannot be evaluated from this single case with a short follow-up. Third, the necessity of the gastro-jejunostomy component versus simpler alternatives (such as lateral esophago-gastric anastomosis with wide pyloroplasty) remains unproven.

### Oncological Safety Considerations and Protocol Modification

This case revealed an important learning point regarding surgical sequence. The initial approach of performing esophago-gastric anastomosis before definitive margin confirmation represented a deviation from standard oncological principles. While endoscopic carbon nanoparticle injection provided preoperative marking, it cannot reliably detect submucosal or subserosal extension, and reconstruction before margin confirmation introduces unnecessary oncological risk.

Following this case, we have modified our surgical protocol as follows: the esophagus is now completely dissected first, with careful gross examination and, when indicated, frozen section evaluation of margins before proceeding with any anastomotic reconstruction. This sequence maintains oncological safety while preserving the technical benefits of our reconstruction method.

This experience underscores the importance of maintaining established oncological principles even when implementing novel techniques, and we recommend this modified sequence for future applications of Itihaas’s Anastomosis.

Total Gastrectomy vs. Proximal Gastrectomy Considerations: Total gastrectomy eliminates reflux entirely but results in complete loss of gastric reservoir function, increased operative morbidity, lifelong nutritional deficiencies (particularly B12 deficiency), dumping syndrome, and significantly impaired quality of life due to eating restrictions. Proximal gastrectomy preserves distal gastric function and reservoir capacity, maintaining some intrinsic factor production and better nutritional outcomes. However, it suffers from severe gastro-esophageal reflux—the primary reason many surgeons default to total gastrectomy. Our Itihaas’s Anastomosis specifically targets this main limitation through three mechanisms: the esophago-gastrostomy creates a neo-fundus that may prevent direct acid contact with the esophagus, the gastro-jejunostomy diverts acid away for neutralization, and the jejuno-jejunostomy prevents bile/pancreatic juice reflux into the gastric remnant.

Proximal gastrectomy is indicated for early-stage gastric cancer in the proximal region of the stomach. However, one of the most discussed technical issues is the method of anastomosis after proximal gastrectomy, primarily to address reflux syndrome, which can irritate the patient and significantly undermine their quality of life post-surgery. The esophageal mucosa is less resistant to the highly concentrated acid secreted by the remaining distal gastric walls. Various anastomosis methods have been introduced over many decades, most aiming to reduce reflux syndrome, an inevitable consequence of proximal gastrectomy [1,2,3].

The simplest anastomosis method is esophago-gastrostomy, which is less complicated, time-saving, and likely involves fewer immediate postoperative complications due to a single anastomotic site [3]. However, it is prone to a much higher rate and severe scale of gastro-esophageal reflux syndrome. There have been debates on the differences in symptoms between anterior and posterior gastric wall anastomosis with the esophagus stump. Simple jejunal interposition between the esophagus and the stomach or double-tract anastomosis (esophago-jejunostomy and jejuno-gastrostomy) are approaches aimed at reducing reflux syndrome by lengthening the distance from the pylorus to the esophagus, thereby decreasing acid reflux into the esophagus [4]. Constructing a tubular cavity of the stomach was proposed earlier [5], and later the “Cheng’s Giraffe” technique (esophago-gastrostomy) was suggested, which similarly involves partial longitudinal resection of the proximal gastric wall (for early-stage tumors at the lesser curvature of the stomach), preserving partial gastric cavity at the greater curvature [6]. However, none of these anastomotic techniques compensate for the lack of the cardia.

Technical Flexibility Within Core Innovation: The key innovation of Itihaas’s Anastomosis lies in the triple anastomosis concept rather than the specific anastomotic techniques employed. While we performed a side-to-side esophago-gastrostomy in this case, a side-to-end esophago-gastrostomy with circular stapler can alternatively be used when anatomically more suitable. The fundamental principle remains the three-anastomosis approach: esophago-gastrostomy (creating the neo-fundus), gastro-jejunostomy (acid diversion), and jejuno-jejunostomy (bile diversion). The specific anastomotic techniques can be adapted to individual anatomy and surgeon preference while preserving the core therapeutic concept. While we lack objective validation of anti-reflux mechanisms, our technique focuses on creating appropriate angulation through side-to-side anastomosis positioning and ensuring adequate intra-abdominal esophageal length. However, specific technical details for optimizing the angle of His recreation require further development and validation through objective studies including manometry and pH monitoring.

Recently, single- or double-flap techniques have been introduced to mimic the cardiac sphincter around the esophagus after esophago-gastric anastomosis [7,8]. However, long-term results on quality of life (QOL) are yet to confirm their efficacy. Consequently, there is a trend to perform total gastrectomy for any stage of gastric cancer to prevent the issues discussed above [1,2]. However, the QOL after total gastrectomy remains a major concern in the field [9]. There is no prospective randomized controlled trial that compares immediate or long-term postoperative complications after proximal gastrectomy. Therefore, all the above methods are considered exploratory anastomosis without definitive results [3,10].

## 3. Conclusions

In summary, Itihaas’s Anastomosis represents a novel technical approach for reconstruction following proximal gastrectomy that theoretically addresses the primary complication of gastro-esophageal reflux. This preliminary case demonstrates technical feasibility with acceptable short-term outcomes. However, all claimed benefits remain theoretical without objective evidence of anti-reflux mechanism recreation. This preliminary technical description requires validation through systematic case series with objective reflux assessment (pH monitoring, manometry, endoscopy), validated quality of life instruments, long-term follow-up, and comparison with established reconstruction methods. We are currently developing such a study protocol. This case report serves as the foundation for future systematic evaluation of this technique.

Comparison with Established Techniques: The abdominal approach for proximal gastric cancer has been validated by the landmark JCOG9502 trial, which demonstrated equivalent survival with reduced morbidity compared to thoracoabdominal approaches [11]. Our technique builds upon this established approach while attempting to address the primary limitation of proximal gastrectomy—gastro-esophageal reflux.

### 3.1. Technical Rationale and Future Directions

The jejuno-jejunostomy component of our technique follows principles similar to the Braun anastomosis, which has demonstrated efficacy in preventing bile reflux in other gastro-jejunostomy procedures. The established success of Braun anastomosis in preventing bile reflux after gastro-jejunostomy, particularly in Billroth II reconstructions and pancreaticoduodenectomy, directly influenced our approach. Our institutional experience with uncut Roux-Y reconstruction informed the design of this anti-reflux strategy [12].

We are currently planning a systematic case series to address these limitations. Future studies will include the following:

- Objective reflux assessment using 24 h pH monitoring;
- Endoscopic evaluation of the neo-fundus anatomy;
- Quality of life assessment using validated instruments;
- Comparison with simpler reconstruction alternatives;
- Long-term nutritional and functional outcomes.

This planned case series will provide the objective validation currently lacking in this preliminary technical description.

### 3.2. Patient Perspective

The medical team proposed a radical proximal gastrectomy, followed by a unique reconstruction method (Itihaas’s Anastomosis) aimed at minimizing acid reflux, a common issue after such surgeries. The three anastomoses—esophago-gastrostomy, gastro-jejunostomy, and jejuno-jejunostomy—were explained to me as crucial steps to ensure my digestive system continued to function well and reduce the risk of reflux.

Post-surgery, my recovery process was carefully monitored. The initial days involved managing mild discomfort and a slight fever, but there were no major complications like infections or anastomotic leaks. By the third day, I was able to start drinking liquids, and gradually I moved on to solid foods. My progress was steady, with no significant pain or digestive issues. A radiological examination confirmed that the reconstructed GI tract was functioning smoothly, with no reflux or obstruction.

The extended hospital stay allowed my doctors to ensure everything was healing correctly, especially given that this was the first time they performed this specific anastomotic procedure. By the 16th day, I was discharged with a positive outlook, reassured by the meticulous care and innovative surgical approach. This experience has been positive in the short term, though I understand that a longer follow-up will be needed to determine the true effectiveness of this reconstruction method.

## Data Availability

The data presented in this study are available within the article. Additional supporting data may be available from the corresponding author upon reasonable request, being subject to patient privacy considerations and institutional policies.
